# Effect of the time interval between oocyte retrieval and ICSI on embryo development and reproductive outcomes: a systematic review

**DOI:** 10.1186/s12958-021-00717-0

**Published:** 2021-03-01

**Authors:** Xue Wang, YaLing Xiao, ZhengYi Sun, JingRan Zhen, Qi Yu

**Affiliations:** Department of Gynecology Endocrine and Reproductive Center, Peking Union Medical College Hospital, Peking Union Medical College/Chinese Academy of Medical Sciences, Beijing, 100730 China

**Keywords:** Oocyte denudation, ICSI, Fertilisation rate, Embryo development, Clinical pregnancy rate

## Abstract

**Background:**

Intra-cytoplasmic sperm injection (ICSI) is used in assisted reproductive technology (ART) laboratories. However, there is no consensus regarding the precise time intervals within ICSI cycles [oocyte pick up (OPU), oocyte denudation (DN), and ICSI], and results are inconsistent and contradictory. Thus, we aim to evaluate whether there is a concordance regarding the time intervals used in different laboratories and a concrete time that gives better laboratory and reproductive results.

**Methods:**

A systematic review of the literature until July 25, 2020, was performed with the keywords “Oocyte Denudation/Denudation/Oocyte,” “Intra-cytoplasmic Sperm Injection/ICSI,” “Oocyte/Oocyte maturation/ cumulus,” and “Cumulus removal/ removal.” Articles and abstracts in English and involving human subjects referring to the effects of oocyte DN time on embryo development and clinical outcomes were included.

**Results:**

Of the 294 evaluated articles, 24 (including 20 full articles and 4 abstracts) were included in this review. Eighteen studies analysed the effect of OPU-DN time on embryo development and clinical outcomes. Most of these studies concluded that OPU-DN time did not influence ICSI outcomes, but some suggested that oocytes should be incubated for a short time before DN to improve oocyte maturity and enhance ICSI outcomes. In addition to reports on positive or negligible effects, adverse effects were reported in 12 studies on DN-ICSI timing. Neither OPU-DN nor DN-ICSI time could improve live birth rate.

**Conclusions:**

Oocytes should be pre-incubated for a short duration (preferably < 4 h) before DN according to the ART laboratory schedule. More randomised controlled trials are warranted to clarify the effect of DN-ICSI timing on ICSI outcomes.

## Background

Intra-cytoplasmic sperm injection (ICSI) is a technique to achieve fertilisation for couples with severe male infertility, in which the sperm is inserted into the cytoplasm of the oocyte [1]. After oocyte collection, the cumulus cells need to be denudated before ICSI, a process called oocyte denudation (DN). However, different assisted reproductive technology (ART) laboratories implement different protocols depending on their daily workload and different time intervals, including the time of oocyte pick up (OPU), DN, and ICSI [2–5].

During follicular development, cumulus cells that surround the oocytes can promote maturation of cytoplasm and nucleus of the oocytes through an autocrine/paracrine mechanism and communicate through gap junction [6, 7]. Before ovulation, cumulus cells proliferate widely and provide basic nutrition for oocytes to support their development. Additionally, cumulus cells also participate in meiotic arrest, transcription regulation, and induction of cytoplasmic maturation [8, 9]. After ovulation, oocytes resume meiotic separation under Leutinizing Hormone (LH) stimulation; they complete the first meiosis, stop at the second meiosis metaphase (MII), and reach meiotic maturation [10]. Simultaneously, cumulus cells produce hyaluronic acid and expand in vitro, and further induce oocyte cytoplasmic maturation by stimulating gene expression and reducing oxidative stress [11, 12]. Research shows that after ovulation induction, gap junctions between cumulus cells around oocytes continue to exist, whereas distant junctions disappear. Using this strategy, intact cumulus cells can use gap junctions between oocytes to promote oocyte maturation [13]. However, DN must be performed prior to ICSI for two reasons: a) the cumulus cells affect the entrance of the injection needle, and that a cumulus-oocyte-complex (COC) cannot be held properly by the holding pipette; b) oocyte maturity must be assessed since only mature oocytes that have reached the MII stage should be injected. Moreover, the polar body has to be placed at 12 or 6 o’clock positions to avoid the oocyte spindle from being damaged [14].

Therefore, an important question arises: When should the cumulus cells around the oocytes be removed to obtain the optimal ICSI outcome? Though many researchers have tried to confirm the optimal timing intervals in ICSI procedures, these time intervals and their impact on the outcomes of ICSI cycles remain controversial. Some studies have found that prolonging the incubation periods between oocyte pickup (OPU) and DN can improve the rates of fertilisation and blastocyst formation, which is conducive to embryo development. It is suggested that oocytes should be incubated for a few hours before DN [15–17]. However, recent studies in mice have shown that prolonged incubation time before DN can induce oocyte apoptosis. In contrast, some studies suggest DN immediately after oocyte retrieval [18]. Other studies found that prolonging the time of OPU-DN could not improve the rates of fertilisation or clinical pregnancy [5, 19]. A negative correlation between the time of DN and ICSI has also been reported, i.e., longer DN-ICSI time lowers the fertilisation rate, and may lead to adverse pregnancy outcomes [17, 20]. Therefore, the aim of this study was to systematically analyse and review the published literature on the impact of time intervals on embryo development and clinical outcomes and determine the optimal time intervals during ICSI cycles.

## Materials and methods

**Fig. 1:**
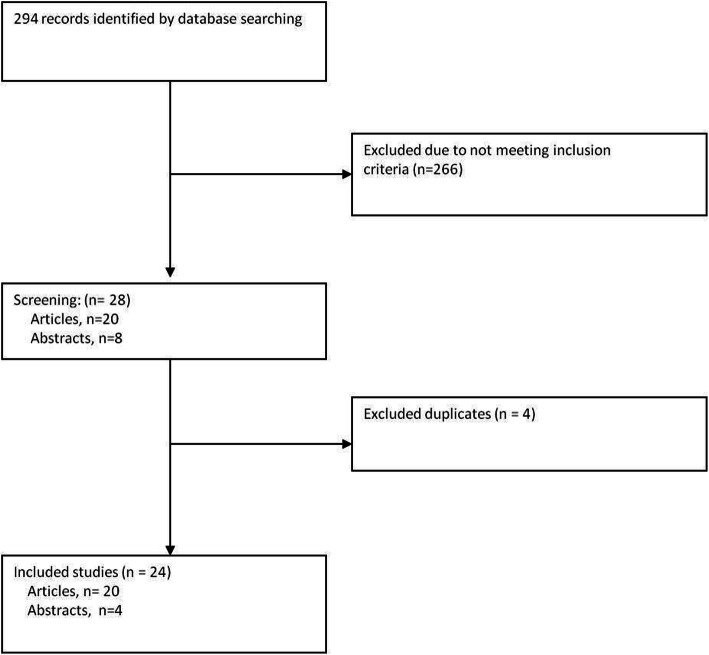
Flow chart for the systematic literature review

PubMed and the Embase databases were searched for relevant studies and reviews using the following keyword combinations: 1) ‘Oocyte Denudation/Denudation/Oocyte’ and ‘Intra-cytoplasmic Sperm Injection/ICSI’ and 2) ‘Oocyte/Oocyte maturation/ cumulus’ and ‘Cumulus removal/ removal.’ The search strategy was limited to articles published in English involving human subjects, with the last search performed on 25 July 2020. Articles that described the effect of DN time on embryo development and clinical outcomes were included in this study, with the additional requirement that they should include at least one of the following indicators: mature egg rate, fertilisation rate, high-quality embryo rate, cleavage rate, blastocyst formation rate, implantation rate, clinical pregnancy rate, and live birth rate. The specific selection process is shown in Fig. 1.

## Results

### Design and methodology of the studies

A total of 294 articles were identified in our initial search, of which 24 (four abstracts and twenty articles) were selected for this review. Among the twenty-four studies, sixteen were retrospective, and only eight were prospective. The characteristics of the studies that we included in the relationship between oocyte DN time and ICSI outcomes are presented in Table 1. Two of these studies utilised donor oocytes [19, 28], and the study of Bárcena et al. [19] included both fresh and freeze-thawed oocytes. Therefore, to avoid the impact of freezing on oocytes, only fresh oocyte data from their study were included. The other 22 studies used autologous oocytes from patients themselves. Three abstracts did not mention the ovarian stimulation protocol [27, 28, 30]. Of the other 21 studies, those prior to 2011 used the GnRH agonist protocol (GnRHa). Later studies used GnRHa and GnRH antagonists (GnRH ant) except for one that only used GnRH ant. Additionally, there has been one study on natural cycles and micro-stimulation protocols [29]. Five studies did not mention the time from human chorionic gonadotropin (HCG) administration to oocyte collection. Most of the other studies used approximately 36 h, with the shortest time being 34 h [5, 26, 35], and the longest time being more than 39 h [34]. Four studies recorded the time with an automatic radiofrequency-based system (RI-Witness, Research Instruments, UK) [19, 20, 32, 35], and the others recorded the time manually. Of the 24 studies, 14 did not mention the environment in which oocytes were incubated, while 7 mentioned 20% O_2_, and only 3 used 5% O_2_ [15, 32, 35]. Eighteen studies analysed the effect of OPU-DN time on embryo development or pregnancy outcomes. Patrat et al. [17] analysed an interval of 0.5 h, whereas Pujol [20] and Bárcena et al. [19] were grouped according to a decile. Six of the studies conducted ICSI immediately after DN. One study was grouped according to HCG-DN [35]. Twelve studies analysed the influence of DN-ICSI time on the outcomes; however, in one study that used a fixed DN time, objects were grouped by OPU-ICSI time [24].

**Table 1:** Basic characteristics of the 24 studies included in the review

Author	Study design	N	Protocol for COH	O_**2**_%	Timing control	HCG-OPU (h)	OPU-DN (h)	DN-ICSI (h)	OPU-ICSI (h)	Grouping criteria
Velde et al. (1998 [21])	PS	60	GnRHa	NM	O	36	1–6	0–4	1–6	Three groups: ①OPU-DN: 1–2 h, DN-ICSI: 0 h or 4 h; ②OPU-DN: 1–2 h and DN-ICSI: 0 h or OPU-DN: 5–6 h and DN-ICSI: 0 h; ③OPU-DN: 1–2 h and DN-ICSI: 4 h or OPU-DN: 5–6 h and DN-ICSI: 0 h
Yanagida et al. (1998) [22]	PS	544	GnRHa	NM	O	> 35	1–11	0	1–11	Grouped by OPU-DN: 1–3 h; 3–5 h; 5–7 h; 7–9 h; 9–11 h
Rienzi et al. (1998) [23]	RS	95	GnRHa	20%	O	36	2–12	0	2–12	Grouped by OPU-DN: ≤3 h; 3–6 h; 6–9 h; 9–12 h
Andrews et al. (2001) [24]	RS	1210	GnRHa	NM	O	36	0–1	Some time	0–5	Grouped by OPU-ICSI: < 3 h; 3–5 h; > 5 h
Hassan et al. (2001) [2]	PS	141	GnRHa	20%	O	NM	0–4	0–4	0–4	Three groups: ①OPU-DN: 0 h and DN-ICSI: 0 h; ②OPU-DN: 4 h and DN-ICSI: 0 h; ③OPU-DN: 0 h and DN-ICSI: 4 h
Jacobs et al. (2001) [25]	RS	432	GnRHa	20%	O	36	> 0–4	0	> 0–4	Grouped by OPU-DN: 0–2 h; 2–3 h; 3–4 h; > 4 h
Ho et al. (2003) [26]	RS	64	GnRHa	NM	O	34	1–8	0	1–8	Grouped by OPU-DN: < 2.5 h; 2.5–3.5 h; 3.5–4.5; 4.5–5.5; ≥5.5 h
Isiklar et al. (2004) [15]	PS	1260	GnRHa	5%	O	36	0–4	0	0–4	Grouped by OPU-DN: 0 h; 2–4 h
Falcone et al. (2008) [3]	RS	135	GnRHa	20%	O	> 36	2	0–10	2–12	Grouped by DN-ICS: 2–4 h; 4–5 h; 5–6 h; 6–7 h; 7–8 h; 9–12 h
Boldi et al. (2010) [27]	RS Ab	203	NM	NM	O	NM	NM	0–3	NM	Grouped by DN-ICS: 0 h; 1–3 h
Aletebi (2011) [4]	PS	309	GnRHant	NM	O	36	0–2	0–2	0–4	Three groups: ①OPU-DN: 0 h and DN-ICSI: 0 h; ②OPU-DN: 1 h and DN-ICSI: 1 h; ③OPU-DN: 2 h and DN-ICSI: 2 h
Patrat et al. (2012) [17]	RS	110	GnRHa	20%	O	36.5 ± 1	0–3	0–3	0–6	Grouped by OPU-DN OR DN-ICSI: 0.5 h as an interval
Esbert et al. (2013) [28]	RS Ab	1212	NM	NM	O	NM	2 or 4	NM	Within 4.5	Grouped by OPU-DN: 2 h; 4 h
Garor et al. (2015) [5]	RS	614	GnRHa/GnRHant	NM	O	34–38	0.08–7	0.08–5.2	NM	Grouped by OPU-DN: < 2 h; > 2 h Grouped by DN-ICSI: < 1 h; > 1 h
Terasawa et al. (2016) [29]	RS Ab	302	MS/NC	NM	O	NM	> 0–3	NM	NM	Grouped by OPU-DN:< 2 h; 2–3 h; > 3 h
Ishikawa et al. (2016) [30]	PS Ab	54	NM	NM	O	NM	0–2	NM	NM	Grouped by OPU-DN: 0 h; 2 h
Pereira et al. (2016) [31]	RS	15	GnRHa/GnRHant	NM	O	35–37	1.9 vs 2.9	NM	NM	OPU-DN was compared between two groups with first complete fertilisation failure and next successful fertilisation in the same patient
Bárcena et al. (2016) [19]	RS	3178	GnRHa/GnRHant	20%	A	Mostly 36	0.5–3	0.6–10.8	1.4–11.7	Ten groups: grouped by OPU-DN OR DN-ICSI: by deciles
Pujol et al. (2018) [20]	RS	1468	GnRHa/GnRHant	20%	A	36	0.4–2.12	0.26–11.19	1.0–12.6	Ten groups: grouped by OPU-DN OR DN-ICSI: by deciles
Mizuno et al. (2018) [16]	PS	54	GnRHa/GnRHant	NM	O	36–38	0 or 2 h	2.5–4	2.5–4.5	Grouped by OPU-DN: 0 h; 2 h
Naji et al. (2018) [32]	RS	2051	GnRHa/GnRHant	5%	A	36	0–5	NM	NM	Grouped by OPU-DN:< 2 h; 2–5 h Subgroup analysis: OPU-DN: 0 h; 0.5–2 h
Zhang et al. (2020) [33]	RS	3602	GnRHa/GnRHant	NM	O	> 36	2.2 ± 0.8	2.8 ± 1	NM	Grouped by DN –ICSI: 0–1 h; 1–2 h; 2–3 h; 3 h–4 h; 4–5 h; > 5 h
Azizi et al. (2020) [34]	PS	613	GnRHa/GnRHant	NM	O	35–39	0.5–6	0–4.8	NM	Grouped by OPU-DN: < 2 h; > 2 h Grouped by DN-ICSI: < 2 h; > 2 h
Maggiulli al. (2020) [35]	RS	1444	GnRHa/GnRHant	5%	A	34–37	2–7	0	2–7	The relationship between blastocyst formation rate and IO-DN was analysed

*RS* Retrospective Study, *PS* Prospective Study, *Ab* Abstract, NM Not Mentioned, *O* operator, *A* Automatically by RI-Witness, *MS/NC* Mini-stimulation /natural cycles, *COH* Controlled Ovarian Hyperstimulation, *HCG* Human Chorionic Gonadotrophin, *OPU* Oocyte Pick up, *DN* Denudation, *ICSI* Intra-cytoplasmic Sperm Injection, *GnRHa* GnRH agonists ,*GnRHant* GnRH antagonists

### Impact of OPU-DN on oocyte maturation rate

The oocyte maturation rate was analysed in 16 studies (Table 2), and 11 studies suggested that prolonging OPU-DN time did not increase the oocyte maturation rate. The other five studies concluded the opposite trend, and one of them found that oocyte maturation was remarkably lower with immediate degranulation than with 4-h incubation with intact cumulus (80.5% vs. 91.9%) [2]. A considerable difference in the oocyte maturation rate was also found between the 2-h and 4-h incubation periods (78.40% vs. 84.81%) [28].

**Table 2:** Effect of OPU-DN time on oocyte maturation and ICSI outcome

Author	Compared times(h)	MOR (%)	FR (%)	GER (%)	BFR (%)	IR (%)	PR (%)	LBR (%)
Velde et al. (1998) [21]	1–2 VS 5–6	N	N	N	ND	ND	ND	ND
Yanagida et al. (1998) [22]	1–3 vs 3–5 vs 5–7 vs 7–9 vs 9–11	N	N	Y	ND	ND	N	ND
Rienzi et al. (1998) [23]	≤3 h vs 3–6 vs 6–9 vs 9–12	N	Y	Y	ND	N	N	ND
Hassan et al. (2001) [2]	0 vs 4	Y	Y	ND	N	ND	N	ND
Jacobs et al. (2001) [25]	0–2 vs 2–3 vs 3–4 vs > 4	N	N	N	ND	N	N	ND
Ho et al. (2003) [26]	< 2.5 vs 2.5–3.5 vs 3.5–4.5 vs 4.5–5.5 vs ≥5.5	Y	N	N	ND	ND	N	ND
Isiklar et al. (2004) [15]	0 vs 2–4	Y	Y	Y	ND	N	N	ND
Aletebi (2011) [4]	0 vs 1 vs 2	Y	Y	ND	ND	ND	N	ND
Patrat et al. (2012) [17]	0 vs 0.5 vs 1 vs 1.5 vs 2 vs 2.5 vs 3	N	Y	N	ND	Y	N	N
Esbert et al. (2013) [28]	2 vs 4	Y	N	N	ND	N	N	ND
Garor et al. (2015) [5]	< 2 vs > 2	ND	N	ND	ND	ND	N	ND
Terasawa et al. (2016) [29]	< 2 vs 2–3 vs > 3	ND	Y	ND	ND	ND	N	ND
Ishikawa et al. (2016) [30]	0 vs 2	N	N	Y	N	ND	ND	ND
Bárcena et al. (2016) [19]	3 h by deciles	ND	N	N	ND	ND	N	N
Pujol et al. (2018) [20]	2.12 h by deciles	ND	ND	ND	ND	ND	N	N
Mizuno et al. (2018) [16]	0 vs 2	N	N	Y	N	ND	N	N
Naji et al. (2018) [32]	0 vs 0–2 vs 2–5	N	N	ND	ND	N	N	N
Azizi et al. (2020) [34]	< 2 vs > 2	N	N	ND	ND	ND	N	ND

*Y* Yes, *N* No, *ND* Not Done, *MOR* Mature Oocyte Rate, *FR* Fertilisation Rate, *GER* Good Embryo Rate, *BFR* Blastocyst Formation Rate, *IR* Implantation Rate, *PR* Pregnancy Rate, *LBR* Live Birth Rate

### Impact of OPU-DN on fertilisation rate and embryo development

Seventeen studies reported data on the effect of OPU-DN on fertilisation rate. Among them, 11 reported no significant effect of OPU-DN time on fertilisation rate, which was similar to the conclusion of a recent study, suggesting no correlation between OPU-DN time and fertilisation rate [33]. However, six studies considered that OPU-DN time did affect fertilisation rate, and five of these believed that the fertilisation rate would increase with the extension of OPU-DN time [2, 4, 15, 17, 23]. Another study found that the fertilisation rate was the highest when oocytes were degranulated within 2 h, compared with that at 2–3 h or longer duration (91.2% vs. 87.3% vs 82.0%, respectively) [29].

The impact of OPU-DN time on embryo development and subsequent embryo quality was evaluated by 11 studies. Among them, six reported no effect on embryo quality. One study was divided into 1–3 h, 3–5 h, 5–7 h, 7–9 h, and 9–11 h groups according to OPU-DN time, and showed that embryo quality that was similar in the first four groups but remarkably higher than that in the fifth group [3]. The remaining study showed a significantly increasing rate of good-quality embryos with extended OPU-DN. Regarding blastocyst formation, two studies found a trend for this to be more frequent for the 2-h or 4-h incubation periods with intact cumulus cells than with immediate degranulation, but the differences were not significant [2, 16].

### Impact of OPU-DN on implantation rate and clinical outcome

A total of seven studies analysed the effect of OPU-DN time on implantation rate. Among them, six concluded that implantation rate was not affected by OPU-DN time, while the remining one found that a group subjected to 1.5–2 h oocyte incubation showed a strikingly higher implantation rate than the other groups [17]. Among the 16 studies on the effect of OPU-DN on pregnancy rate, 15 studies suggested that prolonging OPU-DN time did not lead to any improvement. However, one study found that, compared with the immediate DN group, the clinical pregnancy rate was significantly increased with 1 h or 2 h oocyte incubation prior to DN [2, 4]. The data of the five studies on the effect of OPU-DN time on the living birth rate revealed that there was no significance among groups [16, 17, 19, 20, 32].

### Impact of DN-ICSI on oocyte maturation

Four studies mentioned the effect of DN-ICSI time on the rate of mature oocyte production, three of which found that prolonging DN-ICSI did not improve oocyte maturation [2, 17, 21] (Table 3). One study suggested that prolonging the incubation time before ICSI could promote oocyte maturation, but it was unclear whether the effect originated from the incubation before or after degranulation [4].

**Table 3:** Effect of DN-ICSI time on oocyte maturation and ICSI outcome

Author	Compared times(h)	MOR (%)	FR (%)	GER (%)	BFR (%)	IR (%)	PR (%)	LBR (%)
Velde et al. (1998) [21]	1–2 vs 5–6	N	N	N	ND	ND	ND	ND
Andrews et al. (2001) [24]	< 3 vs 3–5 vs 5–6	ND	N	ND	ND	ND	ND	N
Hassan et al. (2001) [2]	0 vs 4	N	N	ND	N	ND	N	ND
Falcone et al. (2008) [3]	2–4 vs 4–5 vs 5–6 vs 6–7 vs 7–8 vs 9–12	ND	Y	N	ND	ND	Y	ND
Boldi et al. (2010) [27]	0 vs 1–3	ND	N	Y	ND	Y	Y	ND
Aletebi (2011) [4]	0 vs 1 vs 2	Y	Y	ND	ND	ND	Y	ND
Patrat et al. (2012) [17]	0 vs 0.5 vs 1 vs 1.5 vs 2 vs 2.5 vs 3	N	Y	N	ND	N	N	N
Garor et al. (2015) [5]	< 1 vs > 1	ND	N	ND	ND	ND	N	ND
Bárcena et al. (2016) [19]	10.8 h by deciles	ND	N	N	ND	ND	N	N
Pujol et al. (2018) [20]	11.19 h by deciles	ND	Y	ND	ND	ND	Y	N
Zhang et al. (2020) [33]	0–1 vs 1–2 vs 2–3 vs 3–4 vs 4–5 vs > 5	ND	Y	ND	ND	Y	Y	ND
Azizi et al. (2020) [34]	< 2 vs > 2	N	N	ND	ND	ND	N	ND

*Y* Yes, *N* No, *ND* Not done, *MOR* Mature Oocyte Rate, *FR* Fertilisation Rate, *GER* Good Embryo Rate, *BFR* Blastocyst Formation Rate, *IR* Implantation Rate, *PR* Pregnancy Rate, *LBR* Live Birth

### Impact of DN-ICSI on fertilisation rate and embryo development

In terms of fertilisation rate, seven studies considered that DN-ICSI time had no effect on the fertilisation rate, whereas the other five had the opposite conclusions. One study found that the fertilisation rate was negatively correlated with DN-ICSI time (0–3 h) [17]. Another study showed that the fertilisation rate was stable within 6 h after degranulation, and the stability decreased remarkably after 6 h [3], while two others suggested that prolonging the time of DN-ICSI could increase the fertilisation rate [4, 20]. However, one recent study that grouped subjects according to DN-ICSI time found that, although the fertilisation rate gradually increased with incubation times up to 5 h, it decreased considerably after 5 h [33].

Four of the five studies revealed that there was no effect of DN-ICSI time on embryo development. One study showed a non-significant decreasing trend in the frequency of good-quality embryos when the DN-ICSI was more than 5 h [33]. However, another study found more good-quality embryos in a group incubated for 3 h after DN compared to a group with immediate ICSI (37.3% vs. 27.9%; *p* < 0.05) [27]. Only one study on the blastocyst formation rate found that this did not differ significantly between a group incubated for 4 h after DN and a group without incubation after DN (16.7% vs. 18.8%) [2].

### Impact of DN-ICSI on implantation rate and clinical outcome

Three studies analysed the effect of DN-ICSI time on implantation rate. One study found that DN-ICSI time (within 3 h) did not affect implantation rate [17]. One study reported that the implantation rate was 24.6% in the immediate ICSI group, which was considerably higher than that in the 1–3-h incubation group (15.5%) [27]. Another study found that the results varied with different cut-off values of DN-ICSI time. When the cut-off value was 2 h, the implantation rate was strikingly higher in the < 2-h group than in the > 2-h group, but there was no significant difference between the two groups when the cut-off value was 3 or 4 h [33].

A total of ten studies analysed the effect of DN-ICSI time on pregnancy outcomes, half of which indicated that prolonging DN-ICSI time did not improve the clinical pregnancy rate, whereas the other five suggested that DN-ICSI time had an effect. One study found that prolonging DN-ICSI interval duration could improve the clinical pregnancy rate [4], whereas another found that the pregnancy rate increased gradually for the first 6 h, but decreased remarkably afterward [3]. In contrast, one study suggested that longer DN-ICSI duration lowered the clinical pregnancy rate, which found that each 1-h increase in DN-ICSI time reduced the likelihood of clinical pregnancy by 7.9% [14]. Recently, Zhang et al. [33] reported that the clinical pregnancy rate did not vary with DN-ICSI times less than 4 h, but did decrease notably over 4 h. There was no statistical difference in the four studies that analysed the effect of DN-ICSI time on live birth rate [17, 19, 20, 24]. The results and conclusions are listed in Table 4.

**Table 4:** The results and conclusions of the 24 studies included in the review

Study	Items	Results	Conclusions
Velde et al. (1998) [21]	OPU-DN	No difference in MOR, FR, and GER between groups	ICSI should be delayed until the noon hour to observe fertilization in the following morning
	DN-ICSI	No difference in MOR, FR, and GER between groups	
Yanagida et al. (1998) [22]	OPU-DN	The MOR, FR, CR, and PR were similar between groups. When incubated for > 9 h, the MOR was significantly decreased, while the PR showed a downward trend	ICSI is performed at any time within 1–9 h following oocyte retrieval
Rienzi et al. (1998) [23]	OPU-DN	No differences in MOR, CR, and PR between groups. The FR and GER of the group with incubation time of < 3 h were significantly lower than those with incubation time of > 3 h	The optimum time range between 3 and 12 h following oocyte retrieval can improve the fertilization rate and embryo quality
Andrews et al. (2001) [24]	OPU-ICSI	No differences in FR and LBR. The CR of the group incubated for < 3 h was superior to that incubated for > 5 h	The shorter incubation durations (< 3 h) generally seemed to produce better results than longer ones (> 5 h)
Hassan et al. (2001) [2]	OPU-DN	The MOR, FR, BFR, and PR were significantly higher in the 4-h incubation group than those in the immediate DN group	Aspects of nuclear, cytoplasmic maturation, and oolemma properties were improved when oocytes were preincubated with intact cumulus before DN rather than after DN
	DN-ICSI	No differences in FR, BFR, and PR between ICSI group without incubation and those 4 h following incubation.	
Jacobs et al. (2001) [25]	OPU-DN	No significant differences in FR, GER, IR, and PR among all groups	Incubation durations of 30 min to 6 h prior to ICSI did not improve the ICSI results. Should ICSI are needed in advance, no incubation is needed.
Ho et al. (2003) [26]	OPU-DN	The MOR in the group with an incubation time of < 2.5 h was significantly lower than that of > 2.5 h. No differences in FR, GER, and PR among all groups.	Nuclear maturity of the oocytes with hCG administration 34 h earlier could be increased by incubation for 2.5 h before DN
Isiklar et al. (2004) [15]	OPU-DN	The MOR, FR, and GER of 2–4 h incubation group before DN were significantly higher than those of no incubation group. However, no differences in IR and PR between the two groups	Pre-incubation of oocytes prior to ICSI is associated with improved maturation of oocytes, fertilization, and embryo quality
Falcone et al. (2008) [3]	DN-ICSI	With the extension of OPU-ICSI, the FR and CR were gradually increased, peaking at 5–6 h (3–4 h for DN-ICSI), and then gradually decreased with the extension of OPU-ICSI	The most appropriate incubation time for mature oocytes before ICSI is 5–6 h
Boldi et al. (2010) [27]	DN-ICSI	No significant difference in FR between the two groups, however the GER, IR, and PR in the group within 1 h of DN-ICSI were significantly higher than those with 1–3 h of DN-ICSI	Oocytes should be injected as soon as possible following cumulus removal to improve the ICSI outcome
Aletebi (2011) [4]	OPU-DN DN-ICSI	The MOR and FR of the 2-h group were significantly higher than those of the 1-h group; no incubation group before and after DN. The PR of the 2-h group and 1-h group was similar and significantly higher than that of the no incubation group	It is preferable to allow an interval between oocyte retrieval and sperm injection
Patrat et al. (2012) [17]	OPU-DN	The total time of OPU-DN within 3 h had no effect on the MOR and GER. The FR gradually increased, the IR peaked at 1.5–2 h, and the PR reached a high value at approximately 2 h	Incubation of oocytes approximately 2 h before DN may not increase MOR, however may lead to the optimal combination of FR and IR. Meanwhile, the sperm injection should be achieved without any delay following oocyte denudation to maintain good fertilization results
	DN-ICSI	DN-ICSI had no effect on MOR, GER, IR, and PR, however, FR gradually decreased within 3 h.	
Esbert et al. (2013) [28]	OPU-DN	Compared with the 4-h incubation group before DN, the MOR in the 2-h incubation group was significantly lower, while the FR, GER, IR, and PR remained unchanged.	Delaying denudation procedure resulted in a higher MOR, although did not influence clinical outcomes
Garor et al. (2015) [5]	OPU-DN	No impact on the FR and PR regardless of the hCG–OPU interval.	Delaying oocyte denudation or sperm injection did not compensate for insufficient exposure to the follicular environment after hCG triggers before ovulatory oocyte maturation.
	DN-ICSI	No impact on the FR and PR regardless of the hCG–OPU interval.	
Terasawa et al. (2016) [29]	OPU-DN	The FR in the DN group within 2 h of OPU was significantly higher than that in DN group of > 2 h	Cumulus-oocyte complexes are recommended to be denuded soon after OPU in the case of ICSI
Ishikawa et al. (2016) [30]	OPU-DN	No significant differences in the MOR, FR, and BFR between the two groups, however, the rate of high-quality blastocysts was significantly increased after 2 h of incubation	Oocyte culturing with cumulus cells for 2 h or longer improved the resulting blastocyst quality
Pereira et al. (2016) [31]	OPU-DN	The time of OPU-DN in the fertilization group was significantly longer than that in the non-fertilization group	Modulating time intervals between OPU, DN, and ICSI to grant fertilization seems feasible
Bárcena et al. (2016) [19]	OPU-DN	No differences in FR, GER, PR, and LBR among all groups	High-quality oocytes may withstand ageing in vitro to a certain extent, allowing for easier planning of laboratory workflow without a detrimental effect on the outcomes
	DN-ICSI	No differences in FR, GER, PR, and LBR among all groups	
Pujol et al. (2018) [20]	OPU-DN	No effect on PR, continued pregnancy rate and LBR	The PR diminishing was progressive as time OPU-ICSI increases. ICSI should not be delayed whenever possible.
	DN-ICSI	The PR decreased by 7.9%, when the DN-ICSI time was increased by 1 h, with no effect on LBR.	
Mizuno et al. (2018) [16]	OPU-DN	No significant differences in MOR, FR, BFR, PR, and LBR between the two groups, but the high-quality blastocyst rate was significantly increased	Intact cumulus cells should be maintained during the preincubation period, since they are essential to embryonic development post fertilization
Naji et al. (2018) [32]	OPU-DN	No significant difference, in MOR, FR, BFR, IR, PR, and LBR between different groups	Oocyte denudation within 2 h or 2–5 h results in a comparable outcome, permitting more efficiency and flexibility in scheduling laboratory workload
Zhang et al. (2020) [33]	DN-ICSI	The FR increased with longer DN-ICSI interval within 5 h and declined with DN-ICSI interval > 5 h. The PR was significantly higher in DN-ICSI interval of < 4 h compared to that of > 4 h	The optimal time for ICSI is within 4 h following oocyte denudation for excellent outcomes in ICSI cycles
Azizi et al. (2020) [34]	OPU-DN	Be was associated with cytoplasmic granulation and extended PVS of oocytes	The time intervals in the ICSI cycle alters oocyte quality, with no significant impact on the reproductive outcomes
	DN-ICSI	The DN-ICSI was associated with oocytes cytoplasmic granulation. The FR, CR, and PR were not associated with the time intervals in ICSI cycles.	
Maggiulli al. (2020) [35]	IO-DN	No effect on MOR, however, the BFR was decreased while IO-DN increased.	IO-DN did not affect the cumulative live birth rate, but affected the BFR.

*IO* Induction of ovulation, *MOR* Mature Oocyte Rate, *FR* Fertilisation Rate, *CR* Cleavage Rate, *GER* Good Embryo Rate, *BFR* Blastocyst Formation Rate, *IR* Implantation Rate, *PR* Pregnancy Rate, *LBR* Live Birth Rate

## Discussion

For ART to achieve a positive outcome, it is important to obtain high-quality mature oocytes. Oocytes maturation involves both nuclear and cytoplasmic maturation [36]. Nuclear maturation involves recovery from the first meiosis, germinal vesicle breakdown, and the first polar body formation. Cytoplasmic maturation can help prepare the oocyte for fertilisation and subsequent embryonic development, and it can provide enough energy, enzymes, and protein synthesis reserve to meet the needs of new functional protein synthesis during embryonic development [8, 9]. Unlike the nucleus, there is no clear standard for defining and detecting cytoplasmic maturation, which is a highly complex process. In the natural process, the cytoplasm and nucleus may mature synchronously in some way, but they may not be completely synchronised in the ovulation induction cycle [21]. If the cytoplasm of oocytes is not mature during ICSI, this may directly affect fertilisation and embryo development. It may also impair the supply of material to the embryo, resulting in early embryo death and pregnancy failure. Whether intact or incomplete cumulus cells should be incubated before ICSI remains controversial. Therefore, this issue is discussed in the following sections.

After the introduction of ICSI, based on experience with in vitro fertilisation, some researchers hypothesised that incubation of the obtained oocytes before ICSI for a certain duration before DN and/or ICSI might help to achieve a better outcome. However, to date, no consensus has been established. We found that most studies (11/16) concluded that OPU-DN time (0–12 h) did not affect oocyte maturation rate. However, some studies (5/16) showed the opposite trend, which found the oocyte maturation could be improved through a period of incubation [2, 26]. Considering the differences in methodologies used, there may have been certain confounding factors, one of which was the HCG-OPU time. Studies have confirmed that appropriate prolongation of in vivo maturation time, i.e. HCG-OPU > 36 h, can improve oocyte maturation, thereby improving embryo and pregnancy outcomes [37, 38]. In this review, we found that the HCG-OPU time in five studies was less than 36 h [5, 26, 31, 34, 35]. However, in other studies, HCG-OPU was approximately 36 h, with a better homogeneity. Among these five studies, two suggested that prolonging the HCG-OPU and OPU-DN time was beneficial to oocyte cytoplasmic maturation [26, 31]. Ho et al. [26] found that when HCG-OPU was 34 h, the oocyte maturity in the group that were degranulated within 2.5 h was significantly lower than that of other groups, with no differences in fertilization rate and pregnancy outcome. And they suggested that prolonging HCG-OPU time could shorten in vitro incubation [26]. Maggiulli et al. [35] found no correlation between HCG-OPU and mature egg rate and embryo development. Two studies had divided groups into either HCG-OPU < 36 h or > 36 h and found the time of OPU-DN had no effect on fertilization and clinical pregnancy rates regardless of whether HCG-OPU was more or less than 36 h [5, 34]. Therefore, for good homogeneity, we only collected data in groups with HCG-OPU > 36 h in the two studies.

A study has found evidence that meiosis of oocytes might be blocked by the corona-cumulus-complex, suggesting that oocytes could complete meiosis after the removal of cumulus cells [23]. However, almost all the included studies have concluded that DN-ICSI time does not affect oocyte maturation. Hassan [2] compared the effects of OPU-DN and DN-ICSI on oocyte maturation rate, and found that incubation with intact cumulus cells before DN could improve the maturation of oocytes, whereas incubation after DN could not improve the maturation of oocytes. Some studies have suggested that oocyte regulation and the consequent gene expression of cumulus cells and bidirectional control require expression gap junction-associated genes and structural integrity [39]. The expression of connexin and gap junctions was related to oocyte maturation after a short time but decreased with oocyte maturation [40]. Therefore, the role of cumulus cells in controlling oocyte maturation is significant before germinal vesicle breakdown. After ovulation induction, gap junctions between cumulus cells around oocytes continue to exist, whereas distant junctions disappear. Using this strategy, intact cumulus cells can use gap junctions between oocytes to promote oocyte maturation [13]. The specific mechanism underlying this process needs further research.

There is no consensus on the effect of OPU-DN on fertilisation rate; most studies have concluded that there is no significant effect. It has been suggested that this may be due to the synchronous development of the nucleus and cytoplasmic maturation of oocytes during ovulation induction rather than the beneficial effect of peripheral cumulus cells, or that the ICSI may have avoided some mechanisms associated with cytoplasmic maturation [21, 35]. Bárcena et al. [19] have suggested that oocytes from young and fertile people are more tolerant to long-term incubation in vitro without affecting their subsequent development. Patrat et al. [17] suggested that even if the number of mature oocytes could not be increased, incubation would further promote the cytoplasmic maturation of oocytes, improving their fertilisation potential. Peripheral cumulus cells may secrete some paracrine substances and growth factors or express some adhesion molecules to promote the nuclear or cytoplasmic maturation of oocytes [7, 39]. For example, brain-derived neurotrophic factor secreted by cumulus cells is important for the development of the nucleus and cytoplasm of oocytes [41].

Conflicting results concerning the effect of DN-ICSI time on fertilisation were found in the literature. Most authors have concluded that there is no effect, but Patrat et al. [17] have found that DN-ICSI time is negatively related to fertilisation rate. Another study has suggested that a longer incubation time results in spindle instability and chromosomal material loss in oocytes [42]. Therefore, the authors suggested that ICSI should be carried out immediately after degranulation. Some studies found that it was positively correlated with fertilisation rate but significantly decreased from 5 h after degranulation [33]. Some studies suggest that the reason for the increase in fertilisation rate is not only related to the further maturation of cytoplasm, but is also related to oocyte ageing [20, 33]. Oocyte ageing is related to the activities of the M-phase promoter (MPF) and mitogen-activated protein kinase (MAPK), which are important regulators of the second meiosis [43]. With the ageing of oocytes, the activities of MPF and MAPK decrease, which leads to the spontaneous activation of oocytes [44]. Therefore, ageing oocytes with less MPF levels are more likely to be activated by ICSI to form the pronucleus, thus explaining the increase in the fertilisation rate with increased OPU-ICSI time [20].

There is a special situation that cannot be ignored, oocyte spontaneous activation, which is a rapid and uncontrollable process. Normally, oocytes are obtained naturally or COH remains arrested at MII until fertilization, at which the oocyte resumes meiosis [45, 46]. Meiotic arrest is achieved through a series of cytostatic factor activities, such as c-Mos/ MAPK and EMI2 [46–48]. However, in certain cases, advanced maternal age, high FSH exposure for a long time in COH, or high vacuum pressure during oocyte retrieval, may induce oocyte spontaneous activation [49–51]. Oocyte spontaneous activation can lead to premature separation of sister chromatids which are then scattered in the cytoplasm. Once re-activated by a sperm, these separated or scattered chromatids will form 3PN or multiple pronuclei (MPN) [45, 52]. Repeated abnormal fertilization has been previously reported, which may be caused by genetic defect resulting in spontaneous activation [45, 50, 51, 53, 54]. Additionally, the unexplained infertility or repeated pregnancy loss may also be associated with the parthenogenesis caused by spontaneous activation of oocytes prior to ovulation [45, 54]. Therefore, for those who failed to conceive after multiple cycles, especially those who repeat 3PN or MPN, the intervals in ICSI should be avoided since oocyte spontaneous activation could be a contributing factor. To minimize the effect, oocytes should be denuded immediately after collection, followed by a careful and rapid ICSI to mitigate oocyte spontaneous activation-induced abnormal fertilization and possible aneuploidy.

More than half of the studies examined concluded that prolonging OPU-DN time could not improve the rate of good-quality embryos. Some studies found that incubation for 2–4 h before DN could increase the rate of high-quality embryos [15, 16], but that longer times led to a decrease in the rate of high-quality embryos [22]. Underlying reasons include the possibility that cumulus cells may promote the further maturation of oocyte cytoplasm, conducive to subsequent embryonic development. Conversely, COC is sensitive to oxidative stress in vitro, and reactive oxygen species (ROS) can accelerate oocyte ageing, which decreases MAPK activity, meiosis acceleration, the non-separation of sister chromatids, and the increase of aneuploid chromosome number in oocytes [55, 56]. Studies have shown that cumulus cells can secrete glutathione, which may delay the oocyte ageing process and improve embryo quality in a short-term incubation [57, 58]. However, if incubation is prolonged, the antioxidants produced by cumulus cells are not enough to counteract ROS. Therefore, long-term accumulated oxidative stress damages mitochondrial DNA and reduces the ratio of ATP and glutathione/glutathione disulfide in cells, resulting in abnormal cytoskeleton fibres and calcium signalling in the endoplasmic reticulum. It can also induce abnormal calcium oscillation after fertilisation, which manifests as abnormal fertilisation and embryo development [59–61]. One study found that the rate of high-quality embryos in the group performing ICSI immediately after DN was higher than that in the group incubated for 3 h [27]. If the oocytes were incubated for more than 5–6 h, the frequency of high-quality embryos decreased, but the frequency of inferior embryos in the latter three groups was considerably higher than that in the former three groups [3]. The authors suggested that the results indicated that the quality of embryo segmentation depended on the period of oocyte preincubation before injection. In this review, 10 articles had mentioned culture environment, of which only 3 had oocyte incubation at 5% O_2_ [15, 32, 35], while most incubated at 20% O_2_. It has been confirmed that 20% O_2_ can accelerate the formation of ROS in culture medium, induce histone modification, inactivate enzymes, and cause membrane lipid peroxidation, thereby damaging the surface of embryo membrane and affecting its development [56, 62]. Therefore, the obtained oocytes should be incubated in hypoxia environment, which can effectively reduce the production of exogenous ROS and improve the embryonic development potential and outcome of assisted reproduction.

There are a few studies on blastocyst formation rates. Although two studies found that the blastocyst formation rate in the group incubated for 2 or 4 h after DN was higher than that in the immediate degranulation group with no incubation, significant differences were not found [2, 16]. One recent study reported the effect of ICSI immediately after degranulation and after four HCG-DN periods, namely < 37 h, 37–37.5 h, 37.5–38 h, and > 38 h, and found that the blastocyst formation rate decreased with time (44.6% ± 27.5, 39.8% ± 27.2, 36.9% ± 28.4, and 33.0% ± 27.8%, respectively) [35]. They suggested that this was unrelated to the in vitro maturation of immature oocytes, and that the specific mechanism should be studied further [35].

Most of the studies examined for this review have concluded that OPU-DN does not affect the implantation and pregnancy rates. Some found that the highest implantation rate was achieved with 1.5–2 h incubation before DN, a duration that also optimised the pregnancy and live birth rates [17]. The clinical pregnancy rate following incubation for 1, 2, or 4 h before DN was higher than that in the immediate degranulation group [2, 4]. It was suggested that the reason for this could be the appearance of spindles, which resulted from further maturation of the oocyte cytoplasm through the incubation of cumulus cells [4]. It has been confirmed that if the spindles in oocytes are visible upon observation, most of which appears 39–40.5 h after hCG administration, the fertilisation and implantation rates are higher, but after this duration, the oocyte begins to deteriorate. Therefore, it is recommended that ICSI should be performed 39–40.5 h after hCG administration [63, 64]. Considering the effects of the time of DN-ICSI on the pregnancy outcome, Pujol et al. [20] found that, for each 1 h increase in DN-ICSI time, the biochemical and clinical pregnancy rates decreased by 7.5 and 7.9%, respectively, but the continuous pregnancy and live birth rates were unaffected. Moreover, they suggested in vitro ageing of human oocytes significantly affected the chance of becoming pregnant, and ICSI should not be delayed whenever possible [20]. Two studies found that oocytes incubated for 4 h or 6 h resulted in a similar or increased pregnancy rate, which then decreased remarkably [3, 33]. This may be due to the oocytes reaching their best states for fertilisation after incubation within a certain duration before DN. Over-incubation of the oocyte can lead to changes in ultrastructure and gene expression, and increase the incidence of spindle abnormalities, which will affect subsequent embryonic development and reduce the clinical pregnancy rate [63, 65]. Currently, there is no indication that DN time affects the live birth rate.

## Conclusions

According to our literature review on the effects of the time intervals on embryo development and pregnancy outcomes, the results showed that the incubation time before degranulation usually had a small positive effect on ICSI outcome and no negative effect. A short incubation time may be beneficial for embryo development and pregnancy outcome; however, excessive incubation (> 4 h) should be avoided. However, the incubation time after degranulation remains controversial, and negative effects have been observed upon varying this interval, in addition to studies showing no effect or a favourable outcome. Therefore, whether ICSI should be performed after a period of recovery following degranulation should be investigated further. In conclusion, further multicenter, randomised controlled studies with large sample sizes are warranted to optimise the precise timing of the ICSI procedure in the future.

## Data Availability

Data sharing is not applicable to this article as no datasets were generated or analysed during the current study.

## Abbreviations

ART: Assisted reproductive technology
BDNF: Brain-derived neurotrophic factor
COC: Cumulus-oocyte-complex
DN: Denudation
HCG: Human chorionic gonadotropin
ICSI: Intra-cytoplasmic sperm injection
MAPK: Mitogen-activated protein kinase
OPU: Oocyte pick up
ROS: Reactive oxygen species

## References

[1] Palermo G, Joris H, Devroey P, Van Steirteghem AC. Pregnancies after intracytoplasmic injection of single spermatozoon into an oocyte. Lancet. 1992;340(8810):17–8. 10.1016/0140-6736(92)92425-f 1351601

[2] Hassan HA (2001). Cumulus cell contribution to cytoplasmic maturation and oocyte developmental competence in vitro. J Assist Reprod Genet.

[3] Falcone P, Gambera L, Pisoni M (2008). Correlation between oocyte preincubation time and pregnancy rate after intracytoplasmic sperm injection. Gynecol Endocrinol.

[4] Aletebi F (2011). Denudation and sperm injection interval timing: impact on outcome of intracytoplasmic sperm injection. Int J Women's Health.

[5] Garor R, Shufaro Y, Kotler N (2015). Prolonging oocyte in vitro culture and handling time does not compensate for a shorter interval from human chorionic gonadotropin administration to oocyte pickup. Fertil Steril.

[6] Kimura N, Hoshino Y, Totsukawa K, Sato E (2007). Cellular and molecular events during oocyte maturation in mammals: molecules of cumulus-oocyte complex matrix and signalling pathways regulating meiotic progression. Soc Reprod Fertil Suppl.

[7] Richani D, Gilchrist RB (2018). The epidermal growth factor network: role in oocyte growth, maturation and developmental competence. Hum Reprod Update.

[8] Mao L, Lou H, Lou Y, Wang N, Jin F (2014). Behaviour of cytoplasmic organelles and cytoskeleton during oocyte maturation. Reprod BioMed Online.

[9] Watson AJ (2007). Oocyte cytoplasmic maturation: a key mediator of oocyte and embryo developmental competence. J Anim Sci.

[10] Kidder GM, Vanderhyden BC. Bidirectional communication between oocytes and follicle cells: ensuring oocyte developmental competence. Can J Physiol Pharmacol. 2010;88(4):399–413. 10.1139/y10-009 PMC3025001 20555408

[11] Scarica C, Cimadomo D, Dovere L (2019). An integrated investigation of oocyte developmental competence: expression of key genes in human cumulus cells, morphokinetics of early divisions, blastulation, and euploidy. J Assist Reprod Genet.

[12] Dumesic DA, Meldrum DR, Katz-Jaffe MG, Krisher RL, Schoolcraft WB (2015). Oocyte environment: follicular fluid and cumulus cells are critical for oocyte health. Fertil Steril.

[13] Richard S, Baltz JM (2014). Prophase I arrest of mouse oocytes mediated by natriuretic peptide precursor C requires GJA1 (connexin-43) and GJA4 (connexin-37) gap junctions in the antral follicle and cumulus-oocyte complex. Biol Reprod.

[14] Rubino P, Viganò P, Luddi A, Piomboni P. The ICSI procedure from past to future: a systematic review of the more controversial aspects. Hum Reprod Update. 2016;22(2):194–227. 10.1093/humupd/dmv050 26586241

[15] Isiklar A, Mercan R, Balaban B, Alatas C, Aksoy S, Urman B (2004). Impact of oocyte pre-incubation time on fertilization, embryo quality and pregnancy rate after intracytoplasmic sperm injection. Reprod BioMed Online.

[16] Mizuno S, Ishikawa Y, Matsumoto H (2019). The timing of cumulus cell removal for intracytoplasmic sperm injection influences the capability of embryonic development. Reprod Med Biol.

[17] Patrat C, Kaffel A, Delaroche L (2012). Optimal timing for oocyte denudation and intracytoplasmic sperm injection. Obstet Gynecol Int.

[18] Zhu J, Zhang J, Li H (2015). Cumulus cells accelerate oocyte aging by releasing soluble Fas ligand in mice. Sci Rep.

[19] Bárcena P, Rodríguez M, Obradors A, Vernaeve V, Vassena R (2016). Should we worry about the clock? Relationship between time to ICSI and reproductive outcomes in cycles with fresh and vitrified oocytes. Hum Reprod.

[20] Pujol A, García D, Obradors A, Rodríguez A, Vassena R (2018). Is there a relation between the time to ICSI and the reproductive outcomes. Hum Reprod.

[21] Van de Velde H, De Vos A, Joris H, Nagy ZP, Van Steirteghem AC (1998). Effect of timing of oocyte denudation and micro-injection on survival, fertilization and embryo quality after intracytoplasmic sperm injection. Hum Reprod.

[22] Yanagida K, Yazawa H, Katayose H, Suzuki K, Hoshi K, Sato A (1998). Influence of oocyte preincubation time on fertilization after intracytoplasmic sperm injection. Hum Reprod.

[23] Rienzi L, Ubaldi F, Anniballo R, Cerulo G, Greco E (1998). Preincubation of human oocytes may improve fertilization and embryo quality after intracytoplasmic sperm injection. Hum Reprod.

[24] Andrews MM, Fishel SB, Rowe PH, Berry JA, Lisi F, Rinaldi L (2001). Analysis of intracytoplasmic sperm injection procedures related to delayed insemination and ejaculated, epididymal and testicular spermatozoa. Reprod BioMed Online.

[25] Jacobs M, Stolwijk AM, Wetzels AM. The effect of insemination/injection time on the results of IVF and ICSI. Hum Reprod. 2001;16(8):1708-13. 10.1093/humrep/16.8.1708 11473969

[26] Ho JY, Chen MJ, Yi YC, Guu HF, Ho ES. The effect of preincubation period of oocytes on nuclear maturity, fertilization rate, embryo quality, and pregnancy outcome in IVF and ICSI. J Assist Reprod Genet. 2003;20(9):358–64. 10.1023/A:1025476910771 PMC3455840 14531646

[27] Boldi CP, Colasante C, Perego L, De Lauretis L (2010). Effect of early and late oocyte denudation on ICSI outcome. Human Reprod.

[28] Esbert M, Florensa M, Riqueros M, Teruel J, Ballesteros A (2013). Effect of oocyte denudation timing on clinical outcomes in 1212 oocyte recipients. Fertility and Sterility.

[29] Terasawa H, Ueno S, Uchiyama K, Yabuuchi A, Okuno T, Kobayashi T, Kato K (2016). Effect of timing of oocyte denudation after oocyte retrieval on fertilization and pregnancy outcome following cleavage stage single embryo transfer. Human Reprod.

[30] Ishikawa Y, Inaba M, Matsumoto H, Mizuno S, Mori R, Ida M, Fukuda A, Morimoto Y (2016). Influence of the duration between removal of cumulus cells and oocyte retrieval on fertilization and embryonic development. Human Reprod.

[31] Pereira N, Neri QV, Lekovich JP, Palermo GD, Rosenwaks Z (2016). The role of in-vivo and in-vitro maturation time on ooplasmic dysmaturity. Reprod BioMed Online.

[32] Naji O, Moska N, Dajani Y (2018). Early oocyte denudation does not compromise ICSI cycle outcome: a large retrospective cohort study. Reprod BioMed Online.

[33] Zhang Y, Ma Y, Fang Z (2020). Performing ICSI within 4 hours after denudation optimizes clinical outcomes in ICSI cycles. Reprod Biol Endocrinol.

[34] Azizi E, Naji M, Nazarian H (2020). Does timing in ICSI cycle affect oocyte quality and reproductive outcomes? A prospective study. Arch Gynecol Obstet.

[35] Maggiulli R, Cimadomo D, Fabozzi G (2020). The effect of ICSI-related procedural timings and operators on the outcome. Hum Reprod.

[36] Eppig JJ, Schultz RM, O'Brien M, Chesnel F (1994). Relationship between the developmental programs controlling nuclear and cytoplasmic maturation of mouse oocytes. Dev Biol.

[37] Raziel A, Schachter M, Strassburger D, Kasterstein E, Ron-El R, Friedler S. In vivo maturation of oocytes by extending the interval between human chorionic gonadotropin administration and oocyte retrieval. Fertil Steril. 2006;86(3):583–7. 10.1016/j.fertnstert.2006.02.091 16828475

[38] Son WY, Chung JT, Chian RC, Herrero B, Demirtas E, Elizur S, Gidoni Y, Sylvestre C, Dean N, Tan SL. A 38 h interval between hCG priming and oocyte retrieval increases in vivo and in vitro oocyte maturation rate in programmed IVM cycles. Hum Reprod. 2008;23(9):2010–6. 10.1093/humrep/den210 PMC2517153 18556681

[39] Gilchrist RB, Ritter LJ, Armstrong DT (2004). Oocyte-somatic cell interactions during follicle development in mammals. Anim Reprod Sci.

[40] Schramm RD, Bavister BD (1996). Granulosa cells from follicle stimulating hormone-primed monkeys enhance the development competence of in-vitro-matured oocytes from non-stimulated rhesus monkeys. Hum Reprod.

[41] Zhao X, Du F, Liu X (2019). Brain-derived neurotrophic factor (BDNF) is expressed in buffalo (Bubalus bubalis) ovarian follicles and promotes oocyte maturation and early embryonic development. Theriogenology..

[42] Martini E, Flaherty SP, Swann NJ, Payne D, Matthews CD (1997). Analysis of unfertilized oocytes subjected to intracytoplasmic sperm injection using two rounds of fluorescence in-situ hybridization and probes to five chromosomes. Hum Reprod.

[43] Jiang GJ, Wang K, Miao DQ (2011). Protein profile changes during porcine oocyte aging and effects of caffeine on protein expression patterns. PLoS One.

[44] Ebeling S, Labudda A, Meinecke B (2010). In vitro ageing of porcine oocytes: changes in phosphorylation of the mitogen-activated protein kinase (MAPK) and parthenogenetic activability. Reprod Domest Anim.

[45] Combelles CM, Kearns WG, Fox JH, Racowsky C. Cellular and genetic analysis of oocytes and embryos in a human case of spontaneous oocyte activation. Hum Reprod. 2011;26(3):545–52. 10.1093/humrep/deq363 21224285

[46] Cui W, Zhang J, Lian HY, Wang HL, Miao DQ, Zhang CX, Luo MJ, Tan JH. Roles of MAPK and spindle assembly checkpoint in spontaneous activation and MIII arrest of rat oocytes. PLoS One. 2012;7(2):e32044. 10.1371/journal.pone.0032044 PMC3288063 22384134

[47] Madgwick S, Jones KT (2007). How eggs arrest at metaphase II: MPF stabilisation plus APC/C inhibition equals cytostatic factor. Cell Div.

[48] Vogt E, Kirsch-Volders M, Parry J, Eichenlaub-Ritter U. Spindle formation, chromosome segregation and the spindle checkpoint in mammalian oocytes and susceptibility to meiotic error. Mutat Res. 2008;651(1-2):14–29. 10.1016/j.mrgentox.2007.10.015 18096427

[49] Muechler EK, Graham MC, Huang KE, Partridge AB, Jones K. Parthenogenesis of human oocytes as a function of vacuum pressure. J In Vitro Fert Embryo Transf. 1989;6(6):335–7. 10.1007/BF01138772 2534517

[50] Grigoryan H, Levkov L, Sciorio R, Hambartsoumian E. Unexplained total abnormal fertilization of donor oocytes in ICSI with using spermatozoa from different patients. Gynecol Endocrinol. 2019;35(Sup 1):60–2. 10.1080/09513590.2019.1632086 31532319

[51] Ye Y, Li N, Yan X, Wu R, Zhou W, Cheng L, Li Y. Genetic analysis of embryo in a human case of spontaneous oocyte activation: a case report. Gynecol Endocrinol. 2020;36(4):294–6. 10.1080/09513590.2019.1687671 31709844

[52] Cui W, Zhang J, Zhang CX, Jiao GZ, Zhang M, Wang TY, Luo MJ, Tan JH. Control of spontaneous activation of rat oocytes by regulating plasma membrane Na+/Ca2+ exchanger activities. Biol Reprod. 2013;88(6):160. 10.1095/biolreprod.113.108266 23677981

[53] Socolov R, Ebner T, Gorduza V, Martiniuc V, Angioni S, Socolov D. Self-oocyte activation and parthenogenesis: an unusual outcome of a misconducted IVF cycle. Gynecol Endocrinol. 2015;31(7):529–30. 10.3109/09513590.2015.1062861 26137987

[54] Dai J, Leng LZ, Lu CF, Gong F, Zhang SP, Zheng W, Lu GX, Lin G. Time-lapse observation and transcriptome analysis of a case with repeated multiple pronuclei after IVF/ICSI. J Assist Reprod Genet. 2017;34(9):1189–97. 10.1007/s10815-017-0972-9 PMC5581788 28643089

[55] Lian HY, Gao Y, Jiao GZ, Sun MJ, Wu XF, Wang TY, Li H, Tan JH. Antioxidant supplementation overcomes the deleterious effects of maternal restraint stress-induced oxidative stress on mouse oocytes. Reproduction. 2013;146(6):559–68. 10.1530/REP-13-0268 24043846

[56] Belli M, Zhang L, Liu X, Donjacour A, Ruggeri E, Palmerini MG, Nottola SA, Macchiarelli G, Rinaudo P. Oxygen concentration alters mitochondrial structure and function in in vitro fertilized preimplantation mouse embryos. Hum Reprod. 2020;35(6):1476. 10.1093/humrep/deaa047 PMC8453404 32436960

[57] Al-Gubory KH, Fowler PA, Garrel C (2010). The roles of cellular reactive oxygen species, oxidative stress and antioxidants in pregnancy outcomes. Int J Biochem Cell Biol.

[58] Guérin P, El MS, Ménézo Y (2001). Oxidative stress and protection against reactive oxygen species in the pre-implantation embryo and its surroundings. Hum Reprod Update.

[59] Adeoye O, Olawumi J, Opeyemi A, Christiania O (2018). Review on the role of glutathione on oxidative stress and infertility. JBRA Assist Reprod.

[60] Takahashi T, Igarashi H, Amita M, Hara S, Matsuo K, Kurachi H (2013). Molecular mechanism of poor embryo development in postovulatory aged oocytes: mini review. J Obstet Gynaecol Res.

[61] Takahashi T, Igarashi H, Kawagoe J, Amita M, Hara S, Kurachi H (2009). Poor embryo development in mouse oocytes aged in vitro is associated with impaired calcium homeostasis. Biol Reprod.

[62] Bontekoe S, Mantikou E, van Wely M, Seshadri S, Repping S, Mastenbroek S. Low oxygen concentrations for embryo culture in assisted reproductive technologies. Cochrane Database Syst Rev. 2012;11(7):CD008950. 10.1002/14651858.CD008950.pub2 PMC11683526 22786519

[63] Bianchi S, Macchiarelli G, Micara G (2015). Ultrastructural markers of quality are impaired in human metaphase II aged oocytes: a comparison between reproductive and in vitro aging. J Assist Reprod Genet.

[64] García-Oro S, Rey MI, Rodríguez M, Durán Á, Devesa R, Valverde D (2017). Predictive value of spindle retardance in embryo implantation rate. J Assist Reprod Genet.

[65] Trapphoff T, Heiligentag M, Dankert D (2016). Postovulatory aging affects dynamics of mRNA, expression and localization of maternal effect proteins, spindle integrity and pericentromeric proteins in mouse oocytes. Hum Reprod.

